# An integrated analysis of genome‐wide DNA methylation and gene expression data in hepatocellular carcinoma

**DOI:** 10.1002/2211-5463.12433

**Published:** 2018-05-30

**Authors:** Xiang‐Jun Sun, Ming‐Chun Wang, Feng‐Hua Zhang, Xiao Kong

**Affiliations:** ^1^ Linyi People's Hospital China

**Keywords:** DNA methylation, gene expression, hepatocellular carcinoma

## Abstract

Despite progress in the treatment of hepatocellular carcinoma (HCC), 5‐year survival rates remain low. Thus, a more comprehensive approach to explore the mechanism of HCC is needed to provide new leads for targeted therapy. We performed an integrated analysis to discover the relationship between DNA methylation and gene expression in hepatocellular carcinoma (HCC). DNA methylation and gene expression data for HCC were downloaded from The Cancer Genome Atlas (TCGA) database, and differential analysis was performed. Correlation analysis between DNA methylation and gene expression data was then performed in R language. Finally, we selected several crucial genes and evaluated their potential use as diagnostic biomarkers for HCC. In total, 1135 differentially DNA‐methylated CpG sites (DMCs), 377 differentially methylated regions (DMRs), and 1194 differentially expressed genes (DEGs) were identified in HCC. Among the DEGs, 14 genes (*ALX3*,* B4GALNT1*,*CTHRC1*,*DLX5*,*EMX1*,*IRX3*,*OTX1*,*SIX2*,*TLX1*,*VASH2*,*ZIC2*,*ZIC4*,*ZIC5*, and *ZNF695*) exhibited changes in DNA methylation in terms of CpG sites or CpG island (CGI) level, of which *TLX1* and *ZIC4* had the most DMCs (12 and 13, respectively). Further analysis of *CTHRC1*,*ZIC4*,*SIX2*,*VASH2*,*IL17D*,*TLX1*,*OTX1*, and *LART*, examining alterations in both DNA methylation and gene expression level in HCC, showed their potential diagnostic value for HCC was better at the gene expression level than that the DNA methylation level. The DNA methylation status of *CTHRC1*,*VASH2*, and *IL7D* was significantly associated with HCC overall survival (*P*‐value <0.05). This systemic analysis identified a group of novel gene signatures (*CTHRC1*,*ZIC4*, and *OTX1*) that may be regulated by DNA hypermethylation, which may be closely associated with HCC.

**Abbreviations**
CGI: CpG island
DEGs: differentially expressed genes
DMCs: DNA‐methylated CpG sites
DMRs: differentially methylated regions
HCC: hepatocellular carcinoma
TCGA: The Cancer Genome Atlas

Hepatocellular carcinoma (HCC) is one of the most common malignant cancers worldwide, leading to about one million deaths annually 1. HCC is a heterogeneous disease, and the underlying risk factors were liver cirrhosis, alcoholism, chronic hepatitis B virus, and chronic hepatitis C virus. Despite progress in therapy, 5‐year survival rates of HCC remain unfavorable 2. Therefore, it is urgent to deeply explore the mechanism of HCC based on a more comprehensive approach to promote the exploration of targeted therapy.

DNA methylation is a major event of epigenetic modifications which are heritable and stable. Recently, DNA methylation has been extensively investigated in cancer 3. Promoter hypermethylation induces the silencing or downregulation of genes especially for tumor suppressor genes, whereas global DNA hypomethylation leads to genomic instability 4. Emerging evidences have suggested that altered DNA methylation profiles were involved in early stage of HCC 5, 6. Holmila demonstrated that aberrant *VIM* and *FBLN1* methylation levels in cell‐free DNA were potential plasma‐based biomarkers for HCC 7. Nevertheless, DNA methylation alterations in HCC have not been elucidated systemically.

Previous evidences have clearly underscored the crucial role of DNA methylation in the regulation of gene expression in the process of normal development and diseases such as HCC 8. Despite recent advances that permit genomewide screening in the DNA methylation or mRNA expression level, the mechanisms of DNA methylation involved in the regulation of gene expression remain unclear in HCC. The TCGA database offers large‐scale and multigenomic data of over 30 human compressive insight into methylation characters of HCC, we performed differential analysis in DNA‐methylated and gene expression level based on the data from TCGA, and then, a integration analysis of DNA methylation and gene expression changes to uncover the regulatory role of DNA methylation 9. Finally, several crucial genes were selected to validate DNA methylation and gene expression pattern in GEO database and assessed their potential diagnosis value and the correlation with overall HCC survival. Our results may provide some clues for the regulatory role of DNA methylation in HCC.

## Materials and methods

### Data collection

The DNA methylation array data (Illumina Infinium Human Methylation 450 BeadChip), mRNA expression data (IlluminaGA_RNASeqV2.1.0.0), and the corresponding clinical information of HCC were downloaded from TCGA dataset firehose browse (http://firebrowse.org/). According to the corresponding clinical information, the patients with a history of other malignancy were excluded. Consequently, 361 tumor tissues and 41 corresponding adjacent normal tissues from HCC patients were included in this study. According to 361 personal clinical information and methylation data, integrative clinical‐methylation‐expression analysis was performed using R package. Clinical information includes grade, fibrosis, gender, and stage.

### Identification of differentially methylated CpG sites and differentially methylated regions

We removed the CpG sites for which beta value was not available in more than 80% samples, and obtained 395 633 CpG sites. COHCAP package in R 10 was used to analyze the differentially methylated CpG sites (DMCs) and differentially methylated regions (DMRs) between HCC tumor and adjacent normal tissues. Using COHCAP, methylated state and unmethylated state were set, calculating DMRs based on CpG island (CGI) level. Manhattan plot was constructed to explore the distribution of CpG sites according to false discovery rate (FDR) by qqmanpackage in R 11. The top 200 DMCs were used to perform hierarchical clustering analysis by R package pheatmap.

### Identification of differentially expressed genes

Differentially expressed genes (DEGs) between tumor and normal groups were calculated by R package DEseq2 12. FDR was calculated from multiple testing corrections of raw *P*‐value via the Benjamini and Hochberg method 13. Finally, |log2(fold change) | > 2.5 and FDR < 0.01 were set as the threshold for DEGs.

### Construction of weighted comethylation network

After filtering CpG sites that were missing in 80% of samples or the bottom 99% variable CpG sites, we selected 7913 sites to perform WCCNA by WGCNA package in R language 14. The soft threshold was set as 10. The correlation analysis between eigen vectors and tumor–normal state was performed to obtain modules associated with tumor–normal status The threshold was set as| coefficient of correlation |> 0.2 and *P*‐value <0.01. Additionally, CpG sites that were mostly correlated with eigen vectors in modules were selected as hubs. Furthermore, DMC enrichment analysis of modules in comethylation network was performed by Fisher's exact test in R.

### Integrated analysis of DNA methylation and gene expression

To explore the relationship between DNA methylation and gene expression, DMR–DEG pairs were gained in shell. Then, the correlation analysis between DMCs and DEGs was performed in R language and those with |coefficient of correlation | > 0.2 and *P*‐value <0.05 were selected as significant ones.

### Genomic features and functional enrichment analysis

Genomic features included CGI context and gene context. To explore the genomic features of DMC and CpG sites in modules of comethylation network, Fisher's exact test was performed, and those with *P*‐value < 0.01 were considered as significant ones. To discover the function of differentially methylated DEGs and corresponding genes of CpG sites in modules, enrichment analysis was performed by GenoCodis (http://genecodis.cnb.csic.es/analysis) online 15, including Gene Ontology (GO) 16 and Kyoto Encyclopedia of Genes and Genomes (KEGG) pathway 17.

### Receiver operating characteristic curve of candidate genes

To assess the diagnostic value of candidate genes, receiver operating characteristic (ROC) curve was constructed by pROC package in R. Furthermore, area under the curve (AUC) was calculated to assess the performance of each gene.

### Kaplan–Meier analysis of candidate genes

To further explore the relationship of DNA methylation or mRNA expression level of candidate genes with overall survival time of HCC, the Kaplan–Meier curve analysis was performed. A cohort of 378 HCC patients was downloaded from cBioPortal for Kaplan–Meier analysis (http://www.cbioportal.org/). *P* value <0.05 was set as the cutoff of significance; Kaplan–Meier analyses were performed by survival package in R.

### Validation of DNA methylation and expression level of candidate genes

The DNA methylation and expression levels of differentially methylated DEGs were validated through http://www.ncbi.nlm.nih.gov/geo/query/acc.cgi?acc=GSE89852 and http://www.ncbi.nlm.nih.gov/geo/query/acc.cgi?acc=GSE45436 generated from GEO (https://www.ncbi.nlm.nih.gov/) database, respectively, and were analyzed by *t*‐test.

## Results

### The characteristics of DNA methylation pattern in HCC

**Figure 1:**
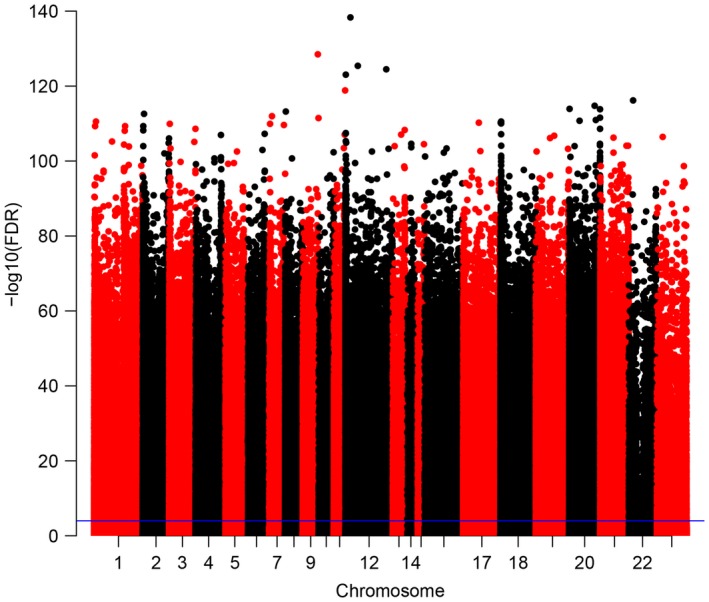
Manhattan plot of CpG sites of HCC. Dots above the purple are CpG sites with FDR < 0.0001.

**Figure 2:**
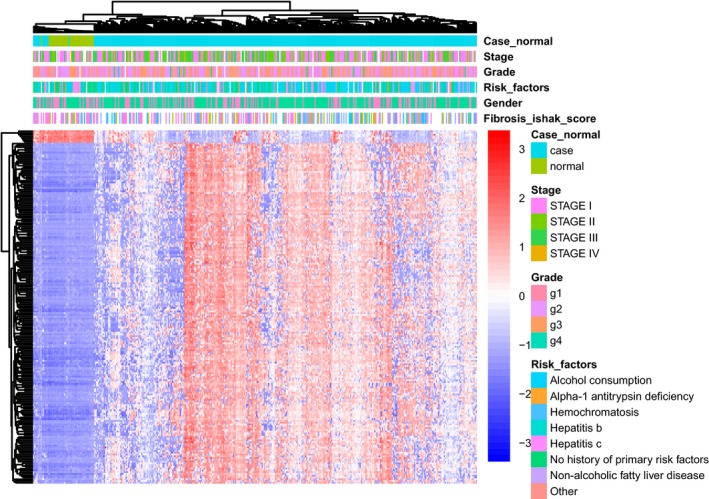
Heat map of top 200 DMCs based on unsupervised hierarchical clustering analysis.

To uncover the methylation profile of HCC, we obtained 227 189 DMCs with FDR <0.0001. As is shown in Manhattan plot (Fig. 1), those CpG sites distributed in all chromosomes. Using the threshold of delta beta >0.2 and FDR <0.0001, we obtained 6510 DMCs totally, including 3903 hypermethylated and 2607 hypomethylated CpG sites (Table S1). Unsupervised hierarchic clustering analysis was performed using the top 200 DMCs based on FDR rank, which showed that the tumor group was in hypermethylated state compared to the normal group (Fig. 2). In table 1, the methylation‐clinical information‐gene expression association result was obtained by setting |correlation|> 0.2 and *P* < 0.05 and integrating with the previous methylation‐gene expression. There were three clinical features including grade, fibrosis, gender which have correlation with the methylation profile.

**Table 1:** Integrative clinical‐methylation‐expression analysis

Methsig	Clinisig	Corsig	P‐valsig	Gene	ID	Cor	*P*‐value
cg00347904	fibrosis_ishak_score	0.202437131	0.003212	–	–	–	–
cg01348293	fibrosis_ishak_score	0.221786909	0.001216	–	–	–	–
cg07254066	fibrosis_ishak_score	0.215263276	0.002383	–	–	–	–
cg09894698	fibrosis_ishak_score	0.211324258	0.002077	–	–	–	–
cg11536474	fibrosis_ishak_score	0.233388754	0.000652	–	–	–	–
cg21790626	fibrosis_ishak_score	0.217589176	0.001512	–	–	–	–
cg22399133	fibrosis_ishak_score	0.248808261	0.000271	–	–	–	–
cg23089825	fibrosis_ishak_score	0.204733884	0.002875	–	–	–	–
cg23817096	fibrosis_ishak_score	0.230638024	0.000758	–	–	–	–
cg24604013	fibrosis_ishak_score	0.217258946	0.001538	–	–	–	–
cg26010734	fibrosis_ishak_score	0.204900751	0.002852	–	–	–	–
cg26149244	fibrosis_ishak_score	0.213574016	0.001855	–	–	–	–
cg26477573	fibrosis_ishak_score	0.205297519	0.002797	–	–	–	–
cg26674943	fibrosis_ishak_score	0.200968278	0.003447	ISL2	64843	0.17766	0.00029
cg27234864	fibrosis_ishak_score	0.22383591	0.001091	IL17D	53342	0.244176	5.24E‐07
cg10073584	gender	‐0.204356353	9.20E‐05	–	–	–	–
cg01348293	grade	0.223914016	2.01E‐05	–	–	–	–
cg07783282	grade	0.210945926	6.04E‐05	–	–	–	–
cg12840719	grade	0.202948974	0.000126	CDKN2A	1029	0.322704	2.43E‐11
cg14175690	grade	0.200063167	0.000148	–	–	–	–
cg14888916	grade	0.22304419	2.16E‐05	–	–	–	–
cg18161327	grade	0.202829167	0.000116	–	–	–	–
cg22167515	grade	0.238598548	5.32E‐06	–	–	–	–
cg22399133	grade	0.204467949	0.000102	–	–	–	–
cg22524657	grade	0.237133078	6.09E‐06	–	–	–	–
cg25340966	grade	0.209457787	6.82E‐05	–	–	–	–
cg25622366	grade	0.203148021	0.000113	OTX1	5013	0.237605	1.07E‐06
cg27234864	grade	0.230871433	1.08E‐05	IL17D	53342	0.244176	5.24E‐07

To gain more robust results, the threshold was set as followings: delta beta>0.2, FDR<0.05, number of DMCs in each CGI ≥ 2. Consequently, 377 significant DMRs on CGI level were obtained, comprising of 359 hypermethylated DMRs and 18 hypomethylated DMRs, which also suggested that the tumor group was more hypermethylated (Table S2).

In terms of genomic features enrichment, DMCs were significantly enrichment in island, shelf, shore on CGI contest level, and gene body, 1st exon, 5′UTR, TSS200, TSS1500, 3′UTR on gene contest level. Specifically, the most enrichment term was gene body (odds ratio = 2.16, 95% confidence interval = 2.06–2.26, *P*‐value = 1.62e‐237) and CGI (odds ratio = 1.97, 95% confidence interval = 1.88–2.07, *P*‐value = 1.10 e‐156).

### Construction of weighted CpG site comethylated network

**Figure 3:**
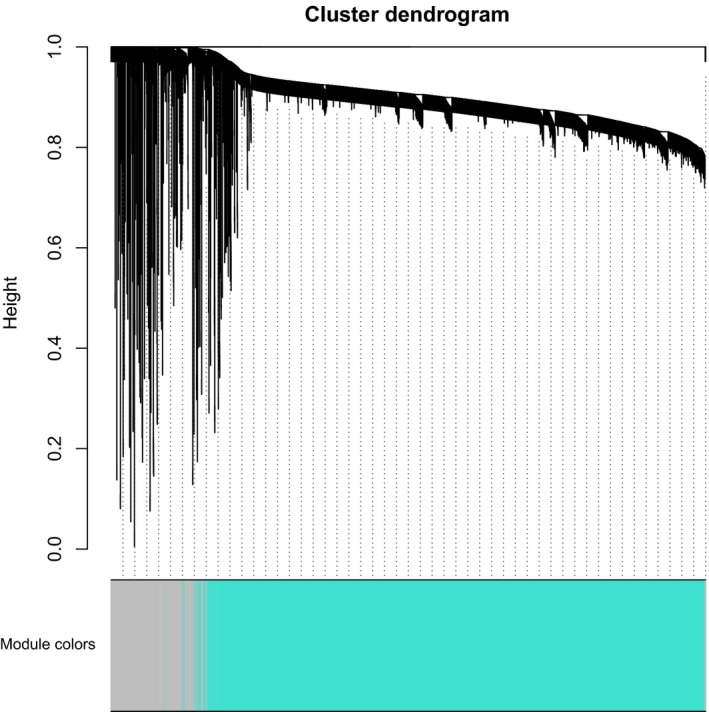
Visualization of the weighted gene co‐expression network analysis. The color row underneath the dendrogram shows the module assignment determined by the Dynamic Tree Cut.

**Figure 4:**
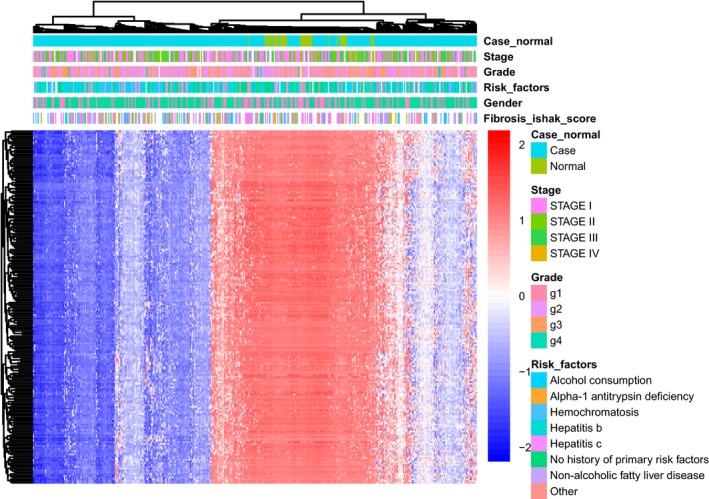
Heat map of top 200 CpG sites detected in turquoise module.

To explore features of comethylated modules of HCC, WCCNA was performed. Consequently, one module named turquoise including 6723 CpG sites was obtained (Fig. 3). This module was significantly associated with tumor–normal status (coefficient value = −0.4, *P*‐value = 2.8e‐17). Enrichment analysis indicated that CpG sites in turquoise module were significantly enriched in DMCs (odds ratio=3.02, 95% confidence interval = 2.68–3.40, *P*‐value = 8.17e‐58). Unsupervised hierarchic clustering analysis showed that the top 200 CpG sites detected in turquoise module apparently clustered all samples into two groups, and the tumor group was in the hypomethylated cluster (Fig. 4). At the methylation‐methylation‐clinical information‐gene expression association analysis, we get no result of methylation‐clinical information.

Furthermore, genomic features enrichment analysis revealed that CpG sites in turquoise module significantly enriched in gene body, 3′UTR, TSS1500, TSS200 on gene context level, and island, shore, shelf on CGI level. The most significant enrichment was in gene body (odds ratio = 6.98, 95% confidence interval = 6.64–7.34, *P*‐value = 0) and island (odds ratio = 0.52, 95% confidence interval = 0.49–0.55, *P*‐value = 2.48e‐109), respectively. Additionally, KEGG pathway enrichment analysis revealed that CpG sites in turquoise module were significantly enriched in neuroactive ligand–receptor interaction, calcium signaling pathway, olfactory transduction, pathways in cancer, basal cell carcinoma, and focal adhesion. Inturquoise module, 10 CpG sites were identified as hubs, which were significantly associated with the module.

### Integrative analysis of differentially DNA‐methylated data and DEGs

To explore the relationship between DNA methylation and mRNA expression, we combined the two‐omics data for further analysis. In brief, 1194 significant DEGs were obtained, consisting of 1017 upregulated genes and 177 downregulated genes (Table S3).

When compared with 377 DMRs on CGI level, 30 DEGs with DMRs on CGI level were obtained (Table 2). There were more than one DMR for *DLX5*,* TLX1*, and *ZIC4*. Moreover, after correlation analysis between DEGs and DMCs, 192 DMC–DEG pairs were obtained, including 132 positive and 60 negative pairs, and 114 DEGs were involved. Among those DEGs, 14 DEGs (*ALX3*,* B4GALNT1*,* CTHRC1*,* DLX5*,* EMX1*,* IRX3*,* OTX1*,* SIX2*,* TLX1*,* VASH2*,* ZIC2*,* ZIC4*,* ZIC5*, and *ZNF695*) exhibited changes in DNA methylation in terms of CpG sites or CpG island (CGI) level, in which *TLX1* and *ZIC4* had the most DMCs (numbers of DMCs were 12, 13).

**Table 2:** Details of 30 DEGs with DMRs on CGI level

CGI	Delta beta	FDR of CGI	ID	Gene	log2(FC)	FDR of gene
chr3:147108511‐147111703	0.224353665	1.5995E‐06	84107	ZIC4	3.901520122	1.55E‐16
chr13:100620241‐100624348	0.224353665	5.06208E‐06	85416	ZIC5	5.929956425	8.56E‐39
chr11:69517840‐69519929	0.224353665	3.55624E‐05	9965	FGF19	2.45740705	0.00000131
chr8:104383409‐104384109	0.224353665	3.72024E‐05	115908	CTHRC1	3.796266642	2.08E‐32
chr8:140714585‐140718259	0.224353665	0.000309591	51305	KCNK9	5.66721426	6.45E‐25
chr16:54317821‐54324604	0.224353665	0.000520355	79191	IRX3	2.504358152	9.91E‐15
chr7:96651963‐96652246	0.224353665	0.000617559	1749	DLX5	4.182961176	1.58E‐15
chr3:147126988‐147128999	0.224353665	0.001253668	84107	ZIC4	3.901520122	1.55E‐16
chr10:102893660‐102895059	0.224353665	0.001305997	3195	TLX1	2.904875821	3.86E‐23
chr2:176986424‐176988291	0.224353665	0.001896195	3235	HOXD9	4.320750438	4.74E‐36
chr7:27212416‐27214396	0.224353665	0.002180964	3206	HOXA10	5.28757278	2.37E‐38
chr12:58021294‐58022037	0.224353665	0.002855985	2583	B4GALNT1	4.392090591	3.69E‐41
chr1:213123647‐213125092	0.224353665	0.003566154	79805	VASH2	2.326558587	5.91E‐17
chr10:102896342‐102896665	0.224353665	0.003643552	3195	TLX1	2.904875821	3.86E‐23
chr2:176993479‐176995557	0.224353665	0.003882799	3234	HOXD8	2.887553258	1.13E‐24
chr15:45421236‐45422394	0.224353665	0.004107949	53905	DUOX1	2.642074122	1.68E‐19
chr2:47796923‐47799166	0.224353665	0.004506572	56660	KCNK12	2.455005376	0.0000154
chr7:96650221‐96651551	0.224353665	0.005157053	1749	DLX5	4.182961176	1.58E‐15
chr7:27225050‐27225629	0.224353665	0.006805565	3207	HOXA11	4.638619017	9.44E‐16
chr7:27225050‐27225629	0.224353665	0.006805565	221883	HOXA11AS	4.73472334	3.43E‐14
chr2:73151200‐73152060	0.224353665	0.009829895	2016	EMX1	4.020354566	2.61E‐23
chr2:177014948‐177015214	0.224353665	0.011476792	3233	HOXD4	5.026837451	2.51E‐24
chr13:100637112‐100637472	0.224353665	0.012182794	7546	ZIC2	6.225385763	7.87E‐69
chr7:96653467‐96654199	0.224353665	0.012301547	1749	DLX5	4.182961176	1.58E‐15
chr1:110610265‐110613303	0.224353665	0.013184706	257	ALX3	2.320617164	7.53E‐17
chr19:1465206‐1471241	0.224353665	0.013184706	10297	APC2	2.150526877	1.89E‐19
chr2:63281034‐63281347	0.224353665	0.015046186	5013	OTX1	4.429178371	3.03E‐29
chr1:247170868‐247171434	0.224353665	0.016182017	57116	ZNF695	4.01893511	1.45E‐16
chr4:13543562‐13546494	0.224353665	0.016772702	579	NKX3‐2	3.81853949	8.22E‐13
chr7:99774733‐99775583	0.224353665	0.019685191	221914	GPC2	2.101347247	1.64E‐12
chr2:26624603‐26625057	0.224353665	0.021209748	92749	C2orf39	2.084461753	0.00000113
chr8:145555342‐145562310	0.224353665	0.022921866	83482	SCRT1	2.8778414	0.000000436
chr10:102891010‐102891794	0.224353665	0.024156614	3195	TLX1	2.904875821	3.86E‐23
chr2:45235511‐45237792	0.224353665	0.033680379	10736	SIX2	5.21721399	9.13E‐29
chr3:40428651‐40429015	0.224353665	0.047131506	956	ENTPD3	2.022142345	0.0000393

In particular, the CpG sites of six DEGs (*SIX2*,* VASH2*,* IL17D*,* TLX1*,* OTX1*, and *LRAT*) involved in DMC–DEG pairs were among CpG sites in turquoise module. Among them, *SIX2*,* VASH2*,* IL17D*,* TLX1*, and *OTX1* were upregulated and hypermethylated. *LRAT* was downregulated and hypermethylated.

### Validation of several crucial genes in other HCC cohorts from GEO database

**Figure 5:**
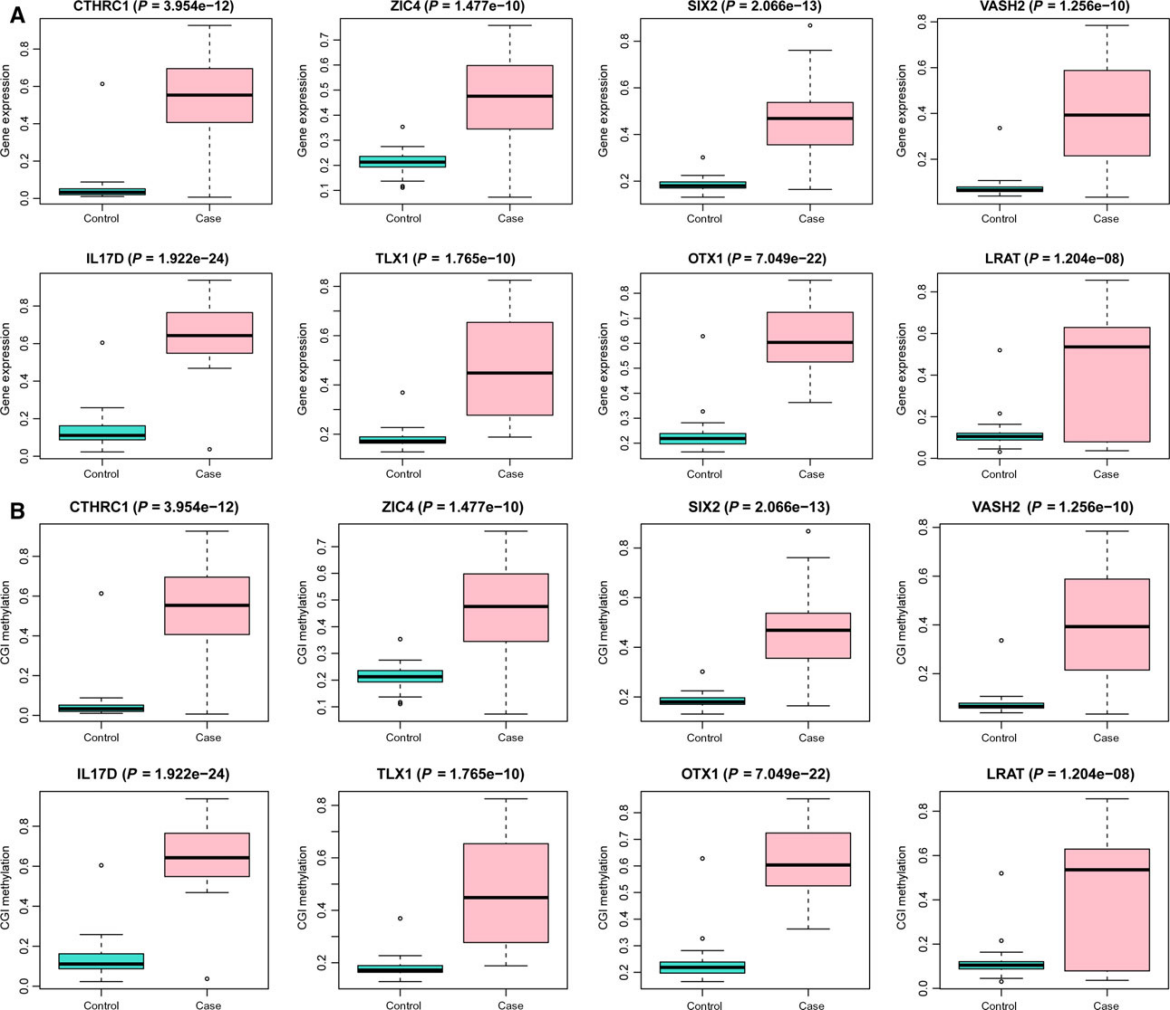
The validation of the methylation and expression levels of candidate genes in HCC tissues compared with nontumor tissues based on GEO database. (A) The methylation level of candidate genes. (B) The expression level of candidate genes.

Based on the above bioinformatics analysis, eight genes (*CTHRC1*,* ZIC4*,* SIX2*,* VASH2*,* IL17D*,* TLX1*,* OTX1*, and *LART*), for which DNA‐methylated status was correlated with their gene expression, were selected for further analysis. To confirm the change in these genes in DNA methylation and gene expression level among HCC patients in other cohorts, http://www.ncbi.nlm.nih.gov/geo/query/acc.cgi?acc=GSE89852 (a dataset presenting DNA methylation data of 74 samples, HCC = 37, normal = 37) and http://www.ncbi.nlm.nih.gov/geo/query/acc.cgi?acc=GSE45436 (a dataset presenting gene expression data of 134 samples, HCC = 93, normal = 41) were used, respectively. The DNA methylation and expression level of the selected eight genes were also significantly changed in the http://www.ncbi.nlm.nih.gov/geo/query/acc.cgi?acc=GSE89852 and http://www.ncbi.nlm.nih.gov/geo/query/acc.cgi?acc=GSE45436 datasets, which was highly consistent with our integrated analysis (Fig. 5A,B).

### Assessment of the potential use of several crucial genes as diagnostic markers of HCC

**Figure 6:**
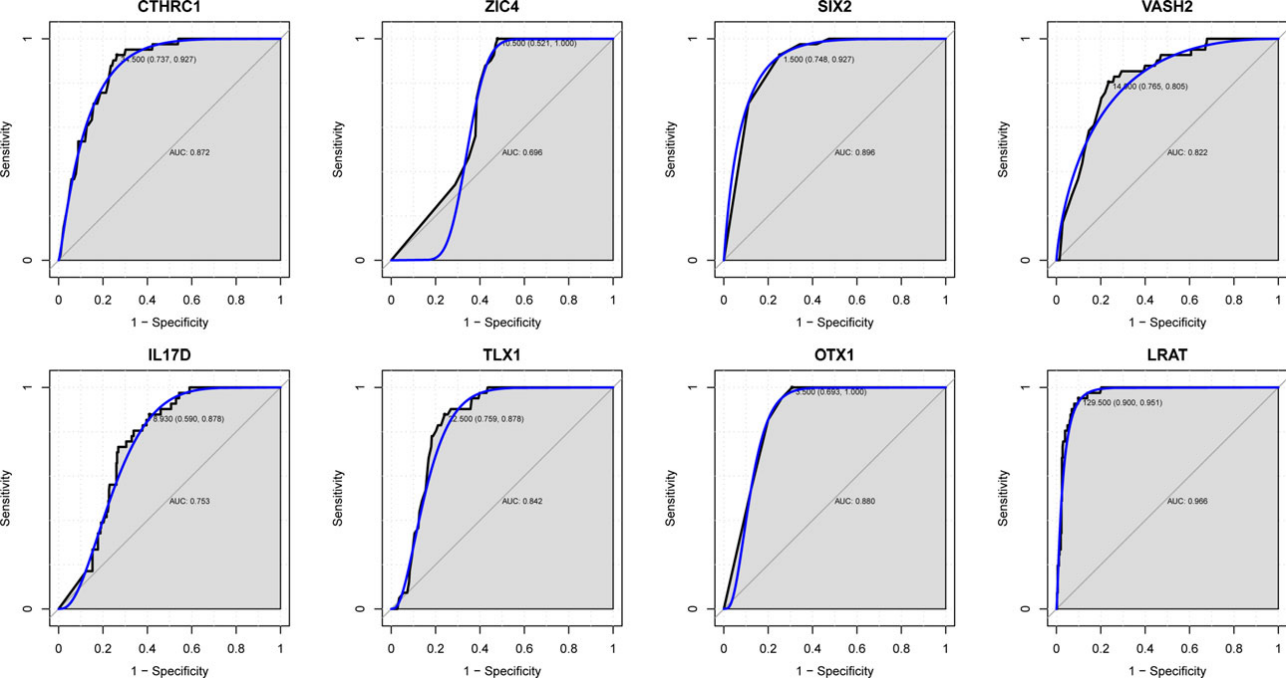
The discriminatory ability of the candidate genes between HCC tissues and adjacent nontumor tissues with ROC curve.

To evaluate their power as diagnostic biomarkers for HCC, we calculated their sensitivity and specificity using ROC curve analysis. For DNA‐methylated patterns of *CTHRC1*,* ZIC4*,* SIX2*,* VASH2*,* IL17D*,* TLX1*,* OTX1*, and *LART*, the AUC was all more than 0.7, with that of *OTX1* showing the most AUC (0.985) (1.000 specificity and 0.973 sensitivity). For gene expression level pattern of the eight genes, the AUC of *CTHRC1* (0.872), *SIX2* (0.896), *VASH2* (0.822), *TLX1* (0.842), *OTX1* (0.880), and *LART* (0.966) was all more than 0.8 (Fig. 6), with that of *LART* showing the most AUC (0.966) (0.900 specificity and 0.951 sensitivity).

### Overall survival analysis by Kaplan–Meier curves in HCC patients

**Figure 7:**
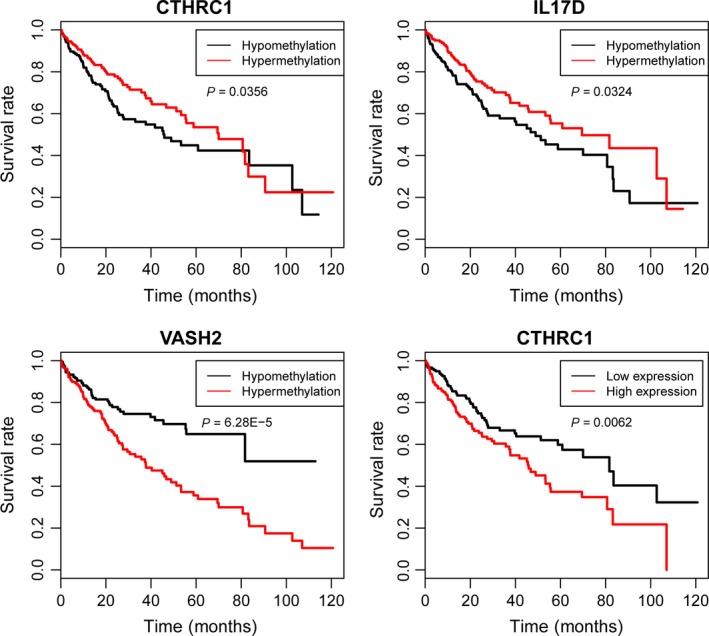
Kaplan–Meier survival curves show the correlation between methylation and expression levels of candidate genes and the overall survival time of HCC.

The correlation between alter of *CTHRC1*,* ZIC4*,* SIX2*,* VASH2*,* IL17D*,* TLX1*,* OTX1*,* LART* in DNA methylated or gene expression level and HCC survival was analyzed through Kaplan–Meier curves. Among them, DNA‐hypomethylated status of *CTHRC1* (*P* = 0.0356) and *IL17D* (*P* = 0.0324) and DNA‐hypermethylated status of *VASH2* (*P* = 6.28E‐5) were significantly associated with poor survival of HCC. Additionally, the upregulation of *CTHRC1* was significantly associated with poor survival of HCC (Fig. 7).

## Discussion

In our study, we characterized DNA methylation profile of HCC by combining the module in comethylated network. Our results showed that HCC tumor group was hypermethylated compared with matched adjacent tissue. CGI is normally unmethylated 18, but our results showed that DMRs were all hypermethylated based on CGI level, indicating that the hypermethylated CGI may affect the expression of corresponding genes. Interestingly, in the DMR–DEG pairs, all the DEGs were hypermethylated and upregulated. Moreover, 69.7% (92 of 132) DMC–DEG pairs were significantly positively correlated.

Moreover, genomic features enrichment analysis indicated that DMCs were significantly enriched in gene body and differentially methylated CGIs in DMR–DEG pairs were all located in gene body. It is well known that CGI hypermethylation in promoter of tumor suppressor genes can drive downregulation of the corresponding genes. This is a classic methylation regulatory pattern in cancer 19. Ju Dong Yang 20 found potential novel oncogenes (*PSRC1*,* MRE11A*,* MYO1E*), tumor suppressor genes (*CFH*,* MYRIP*), implicated in hepatocarcino‐genesis that may be regulated by CpG site methylation, and affect prognosis after resection for HCC. David A. Wheeler 21 found significantly mutated genes, including *LZTR1*,* EEF1A1*,* SF3B1*, and *SMARCA4*, a p53 target gene expression signature correlating with poor survival. Potential therapeutic targets for which inhibitors exist include WNT signaling, *MDM4*,* MET*,* VEGFA*,* MCL1*,* IDH1*,* TERT*, and immune checkpoint proteins CTLA‐4, PD‐1, and PD‐L1. Aberrant DNA methylation in gene body has not been elucidated clearly. In bladder and colon cancer, hypermethylated CpG‐rich regions along with altered expression were detected in gene body of *PAX6* 22. Expressed genes were hypermethylated in active X chromosome regions compared with the corresponding silenced genes 23. Yang *et al*. 24 demonstrated that the hypermethylation status in gene body promoted corresponding gene expression.So hypermethylated CGI in gene body along with upregulated gene expression may provide another DNA methylation regulatory pattern in HCC.

In WCCN, CpG sites in turquoise module were negatively correlated with tumor–normal status, and the unsupervised hierarchical clustering analysis of above CpG sites suggested a hypermethylation status of normal group. This could provide us a methylation characteristic of HCC in a systematical view. The CpG sites enriched in gene body, CGI, and DMCs suggested that there were also some common traits between differential methylation and WCCN analysis.

Moreover, a series of potentially epigenetic regulated genes were identified. *CTHRC1* (collagen triple helix repeat containing‐1), an extracellular matrix‐related secreted glycoprotein, was ubiquitously upregulated in many cancer types. For instance, it has been reported that *CTHRC1* was upregulated by promoter demethylation in gastric cancer 25. In our present study, *CTHRC1* was hypermethylated at CGI level along with downregulated expression. The methylation level of *CTHRC1* acted as a protective factor in HCC, whereas a contrary trend was observed for the expression of *CTHRC1* in our analysis. *CTHRC1* played an important role in attenuating fibrosis in hepatic fibrosis 26. Tameda *et al*. showed that knocking down of *CTHRC1* can suppress cell proliferation and motility in HCC cells. Moreover, the protein of CTHRC1 was significantly overexpressed in poorly differentiated HCC 27. In addition, CTHRC1 activated Wnt/PCP signaling pathway, which promoted cervical cell migration, invasion, and epithelial‐to‐mesenchymal transition (EMT) in renal cell carcinoma and glioblastoma cells 28, 29, 30. Herein, we give additional evidence that upregulation of CTHRC1 may be regulated by aberrant methylation in HCC.

As one member of the zinc finger of the cerebellum family, Zic family member 4 (*ZIC4*) was hypermethylated and was involved in lymph node in oral cancer 31. *ZIC4* was also associated with progression to muscle‐invasive disease in bladder cancer 32. In addition, orthodenticle homeobox 1 (*OTX1*) was upregulated and associated with high stage of colorectal cancer 33. Silencing of *OTX1* suppressed the proliferation and migration by promoting cell cycle arrested in HCC 34. Herein, *ZIC4* and *OTX1* were all hypermethylated on CGI level and upregulated, implying that DNA methylation alteration may play important roles in HCC.

## Conclusion

Briefly, this integrated analysis of genomewide DNA methylation and gene expression data identified a group of novel gene signatures (*ALX3*,* B4GALNT1*,* CTHRC1*,* DLX5*,* EMX1*,* IRX3*,* OTX1*,* SIX2*,* TLX1*,* VASH2*,* ZIC2*,* ZIC4*,* ZIC5*, and *ZNF695*), which may be regulated by DNA methylation in HCC. Our further analysis of *CTHRC1*,* ZIC4*,* SIX2*,* VASH2*,* IL17D*,* TLX1*,* OTX1*, and *LART* showed the potential use of some genes as diagnostic or prognostic biomarkers.

## Author contribution

XS and MW designed and performed the train of thought. FZ analyzed the resulting data, and XK contributed the analysis tools. All authors wrote the manuscript. All authors read and approved the final manuscript.

## Supporting information


**Table S1.** List of 6510 differentially DNA‐methylated CpG sites (DMCs).

**Table S2.** List 377 differentially methylated regions (DMRs).

**Table S3.** List of 1194 differentially expressed genes (DEGs).
